# Metabolites Isolated from *Senecio nutans* Sch. Bip and Their Synthesized Oximes Inhibit Angiotensin I-Converting Enzyme Activity in Vascular Smooth Muscle

**DOI:** 10.3390/ijms26083786

**Published:** 2025-04-17

**Authors:** Javier Palacios, Carlos Villarroel, Daniel Asunción-Alvarez, Fredi Cifuentes, Adrián Paredes, Chukwuemeka R. Nwokocha, Alejandro Castro-Álvarez, Claudio Parra

**Affiliations:** 1 Laboratorio de Bioquímica Aplicada, Facultad de Ciencias de la Salud, Universidad Arturo Prat, Iquique 1110939, Chile; carlosvicentevillaroeldiaz92@gmail.com (C.V.); hasuncion@unitru.edu.pe (D.A.-A.); 2 Laboratorio de Fisiología Experimental, Instituto Antofagasta, Universidad de Antofagasta, Antofagasta 1271155, Chile; fredi.cifuentes@uantof.cl; 3 Departamento Biomédico, Facultad de Ciencias de la Salud, Universidad de Antofagasta, Antofagasta 1240000, Chile; 4 Laboratorio de Productos Naturales, Departamento de Química, Facultad de Ciencias Básicas, Universidad de Antofagasta, Antofagasta 1270300, Chile; adrian.paredes@uantof.cl; 5 Laboratorio de Química Biológica, Instituto Antofagasta, Universidad de Antofagasta, Antofagasta 1271155, Chile; 6 Department of Basic Medical Sciences Physiology Section, Faculty of Medical Sciences, The University of the West Indies, Mona Campus, Kingston 7, Jamaica; chukwuemeka.nwokocha@uwimona.edu.jm; 7 Departamento de Ciencias Preclínicas, Facultad de Medicina, Universidad de La Frontera, Av. Francisco Salazar 01145, Temuco 4780000, Chile; alejandro.castro.a@ufrontera.cl; 8 Departamento de Química Orgánica, Facultad de Ciencias Químicas, Universidad de Concepción, Edmundo Larenas 129, Concepción 4070371, Chile

**Keywords:** acetophenone, arterial hypertension, medicinal plants, molecular docking, oximes, vascular response

## Abstract

Angiotensin-Converting Enzyme (ACE) plays a pivotal role in the renin–angiotensin system, modulating blood pressure and electrolyte homeostasis by deactivating bradykinin and activating angiotensin II. Metabolites from *Senecio nutans* (**1** and **3**), a plant indigenous to the Andean region of the Atacama Desert, and their respective oximes, **2** and **4**, were subjected to molecular docking analysis, employing six ACE crystal structures. ACE activity assays revealed that oximes exhibited superior inhibitory effects compared to metabolites. Among the compounds investigated, **2** emerged as the most potent ACE inhibitor (**2** = 11.5 μM and **4** = 13.4 μM). The vascular contractile response to Angiotensin I showed significant (*p* < 0.05) reductions in Ang I contraction with **2**, **3**, and **4** (97 ± 6%, 81 ± 6%, 81 ± 3% compared to control), while **1** exhibited no such effect. These results reinforce the potential of **2** as a promising ACE inhibitor and highlight its impact on vascular contractility. As such, it is a promising candidate for ACE inhibition and hypertension treatment.

## 1. Introduction

Arterial hypertension is defined as a metabolic disorder characterized by high pressures in the arteries. Its impact is of global significance, as it affects approximately 1.28 billion people, especially those between 30 and 79 years of age [1]. This disease can be triggered by various risk factors, such as age, race, family history, overweight/obesity, lack of physical activity, smoking, excessive salt consumption, potassium insufficiency, stress, alcoholism, and other chronic diseases [2]. High blood pressure presents acute complications, such as hypertensive emergencies, and in the long term, it affects all organs of the body, especially the heart. This vital organ can suffer wear and tear due to excessive effort, increasing the risk of diseases such as ischemia, unstable angina pectoris, and acute myocardial infarction, among others [3]. In cardiovascular physiology, hydroelectrolytic balance and cellular function largely depend on the renin–angiotensin system (RAS), which plays a crucial role. However, its excessive activation contributes significantly to the development of hypertension [4]. Angiotensin-Converting Enzyme (ACE), a key regulator of the RAS, deactivates bradykinin and activates angiotensin II, thus influencing blood pressure and electrolyte homeostasis [5]. ACE inhibitors, like captopril, enalapril, fosinopril, and ramipril, are widely used in chronic management of cardiovascular conditions. However, some ACE inhibitors have limitations, including susceptibility to proteolytic degradation, leading to adverse effects [6,7].

In this context, the development of new drugs is typically inspired by natural products isolated from plants. In the last two decades, about 50% of the drugs introduced on the market have been derived from natural sources [8]. In this sense, one of the medicinal plants used for these purposes is *Senecio nutans* Sch. Beep. from Arid Andean Region of Chile. In previous studies, our research group has reported that the hydroalcoholic extract of this plant can reduce blood pressure in normotensive and hypertensive rats [9]. This effect was due to a protective action on the heart [9,10], but also to a reduction in vascular tone of the rat aorta [11]. The main compounds of this plant, such as prenyl acetophenone and benzophenone derivatives [9,10,11], have demonstrated interesting vasodilatory properties [11], which makes them ideal compounds to use as starting material to synthesize oximes. In recent years, oximes have gained interest, especially as nitric oxide donors and potential antagonists of β_1_ and β_2_ adrenergic receptors [12,13].

We have recently reported that oxime derivatives obtained from *S. nutans* metabolites cause vasorelaxation through an endothelium-dependent pathway [14,15]. Although chemical modifications aim to improve the biological activity of a starting compound, this is not always achieved. An example of this is that the oxime of the acetophenone derivative [15], but not the dihydrobenzofurane derivative [14], enhanced the blocking effect on the voltage-gated L-type calcium channel (LTCC) in the rat aorta. To date, there are no reports on the interaction of *S. nutans* metabolites and their oximes with ACE both in vitro and in silico. In this sense, this work aimed to isolate the main compounds of *S. nutans*, synthesize their respective oximes, and determine their in vitro inhibition and in silico interaction with six crystal structures of Angiotensin-Converting Enzyme.

## 2. Results and Discussion

### 2.1. Determination of the Enzymatic Activity of ACE

**Figure 1:**
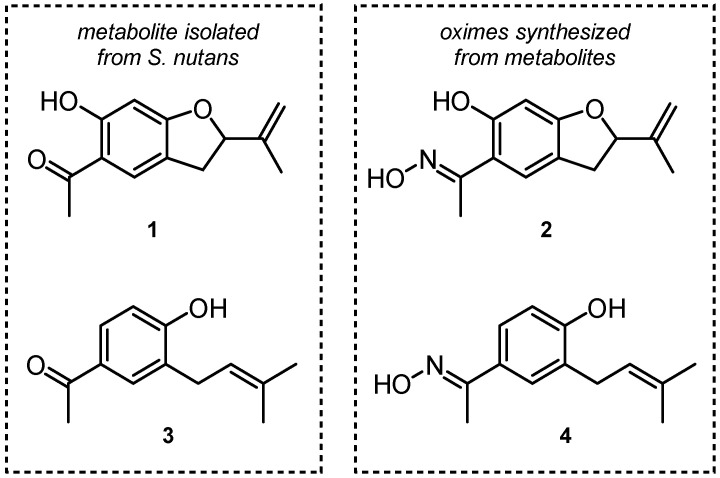
Metabolites isolated from *Senecio nutans* Sch. Beep. 5-acetyl-6-hydroxy-2-isopropenyl-2,3-dihydrobenzofuran (**1**) and 4-hydroxy-3-(3-methyl-2-butenyl) acetophenone (**3**) and their respective oximes (**2** and **4**).

As mentioned above, angiotensin I (Ang I) is a key component in the renin–angiotensin system (RAS), which plays an important role in the regulation of blood pressure, electrolyte homeostasis, and vascular remodeling [16]. Renin produces Ang I by cleaving amino acids from angiotensinogen; then, ACE hydrolyzes Ang I to angiotensin II, which stimulates aldosterone release from adrenal cortex, leading to an increase in blood pressure by sodium reabsorption in the kidneys [16]. In this sense, a screening was used to evaluate *S. nutans* compounds as potential ACE inhibitors (Figure 1). It is important to remember that these compounds showed vascular relaxation in the rat aorta [11,15], so it was interesting to know whether this functionality is associated with ACE activity.

**Figure 2:**
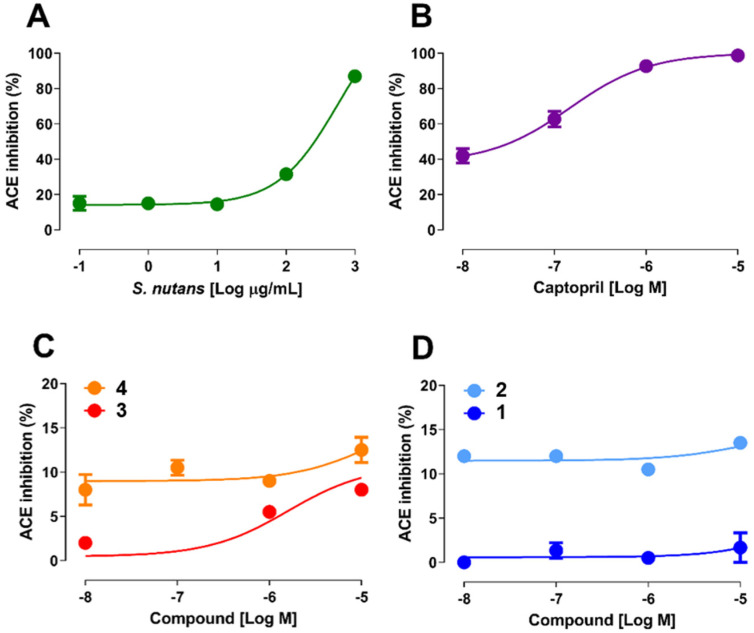
Graph showing the percentage of inhibition of ACE activity. Activity was determined in the presence of *S. nutans* extract (**A**) and different compounds in increasing concentrations (10^−8^ to 10^−5^ M) compared to the positive control captopril (10^−8^ to 10^−5^ M; (**B**)). (**C**) compares **3** and its oxime, while (**D**) is compared to **1** and its oxime. Values represent the mean ± relative standard error (3–4 experiments).

The results showed that *S. nutans* extract significantly inhibited ACE activity by 32 ± 1% and 87 ± 3% (*p* < 0.05), at 100 and 1000 µg/mL (*p* < 0.001), respectively (Figure 2A). Although the pure compounds inhibited ACE activity less than the extract, 8 ± 1% **3** and 13 ± 1% **4** at 10^−5^ M, the inhibition of **4** was significantly higher than that of **3** (Figure 2C). In fact, the affinity to ACE significantly (*p* < 0.001) decreased in the presence of **4** (Km 13.4 µM) versus **3** (Km 1.6 µM). Also, **2** inhibited ACE activity by 14 ± 0.5% at 10^−5^ M (Figure 2D). The Km to ACE for **2** was 11.5 µM. However, **1** did not inhibit ACE activity at all: 2 ± 2%. Interestingly, the inhibitor effect of oximes and captopril occurred immediately at low concentrations (10^−8^ M). On the other hand, the effects of captopril, **3**, and the extract increased in a dose-dependent manner. According to the results of ACE activity, it is plausible to state that oximes presented a higher activity inhibition of ACE activity than metabolites.

ACE activity assays revealed that oximes had stronger inhibitory effects than metabolites. However, the overall inhibition efficiency of the compounds (Figure 2C,D) studied was relatively small compared to captopril, which achieved total inhibition at a log concentration of −5 (Figure 2B). Conversely, the extract of *S. nutans* proved to be even more potent than the metabolites investigated (compounds **1** and **3**), although compound **1** acted contrary to the desired effect. This finding suggests the possibility of synergy among the metabolites or some yet unidentified compound. This is consistent with our previous study, which showed that in rat aorta precontracted with phenylephrine, the relaxation induced by a submaximal concentration of *S. nutans* extract was significantly greater than that of its metabolites (**1** and **3**) [17].

### 2.2. Effect of S. nutans and Pure Compounds on the Vascular Contractile Response to Angiotensin I

**Figure 3:**
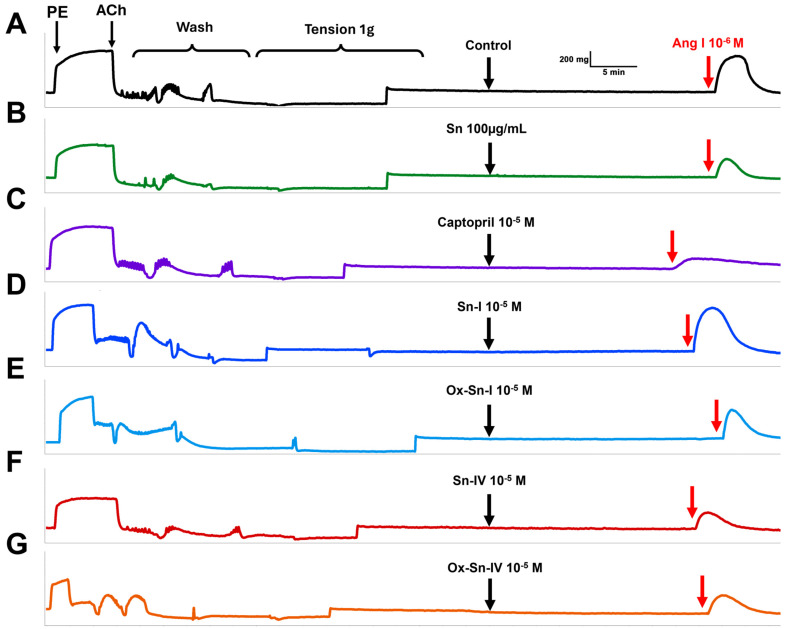
Effect of *S. nutans* and pure compounds on the vascular contractile response to Angiotensin I. The aortic rings were preincubated with the vehicle for 20 min (Control; (**A**)), *S. nutans* extract (Sn; 100 µg/mL; (**B**)), captopril (10^−5^ M; (**C**)), **1** (10^−5^ M; (**D**)), **2** (10^−5^ M; (**E**)), **3** (10^−5^ M; (**F**)), **4** (10^−5^ M; (**G**)), and then angiotensin I (Ang I; 10^−6^ M) was added to the bath. Previously, aortic rings precontracted with PE (10^−6^ M) and relaxed with ACh (10^−5^ M) were added to the bath to evaluate the vascular endothelium. The *X* axis means time (min) and the *Y* axis represents the force of contraction (g).

**Figure 4:**
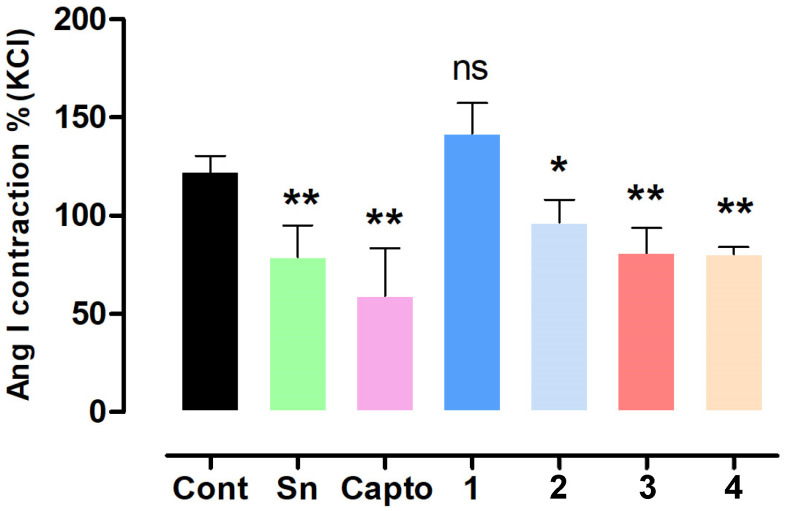
*S. nutans* and their pure compounds reduced the contractile response to angiotensin I (Ang I; 10^−6^ M). The aortic rings were pre-incubated for 20 min with *S. nutans* extract (Sn; 100 µg/mL) and different compounds (10^−5^ M) compared to the positive control captopril (Capto; 10^−5^ M). Values represent the mean ± relative standard error in duplicate of 4–5 experiments. * *p* < 0.05, ** *p* < 0.01 vs. Control. ns: not significant.

Since ACE inhibition is a potential approach for drug targeting in the treatment of hypertension [18], the contractile response to Ang I was evaluated with several compounds from *S. nutans* and captopril as a positive control. The results showed that the preincubation of aortic rings with *S. nutans* extract significantly (*p* < 0.001) reduced the contractile response to Ang I (10^−6^ M): 122 ± 3% control versus 79 ± 6% extract (Figure 3 and Figure 4). Ang I is known to cause vasoconstriction due to the conversion of Ang I to Ang II by the enzymatic catalysis of ACE in the vascular endothelium [19]. ACE activity in the rat aorta is approximately 65% due to the vascular endothelium [17].

Interestingly, a significant reduction in Ang I contraction was found with **2**, **3**, and **4**, at 97 ± 6%, 81 ± 6%, and 81 ± 3% compared to control, while we did not find it with **1** (Figure 4). We expected that the chemical modification of the metabolites (**1** and **4**) would enhance the decrease in vascular contraction to Ang I in the rat aorta. However, it occurred only in **2**, whereas with **4**, the effect was similar to that of **3**. These findings were consistent with the molecular docking analysis. ACE inhibitors enhance the bioavailability of endothelial nitric oxide (NO) because they prevent the degradation of NO by angiotensin II-induced reactive oxygen species (ROS) [20,21].

### 2.3. Molecular Docking Validation

Six crystal structures of Angiotensin-Converting Enzyme (ACE) that contained ligands bound to the enzyme’s catalytic site were used. These complexes exhibited similar structural features in response to interaction with various binding site residues, such as carboxylic acids, peptide bonds, and aliphatic chains. These characteristics were shared by most of the co-crystallized ligands.

The grid fitting parameters were based on the reference to the ligand co-crystallized on the protein. First, the grid was centered relative to the center of mass of the ligand. Then, the grid size was established based on the size of the ligand. Once these parameters were obtained, a self-docking process was carried out to evaluate the reliability of the positioning prediction and identify the need to adjust any parameters necessary to carry out the molecular docking.

The self-docking results were satisfactory, with an RMSD value equal to or less than 2.0 Å, even though there were high values (greater than 1.0), such as the poses achieved for 6EN5, 6F9U and 7Q24. Consequently, these side chains’ greater freedom of movement results in a different arrangement than that of the reference crystal structure. Despite the above, the values achieved validate the coupling protocols proposed for each case (Table 1).

### 2.4. Molecular Docking Analysis

**Figure 5:**
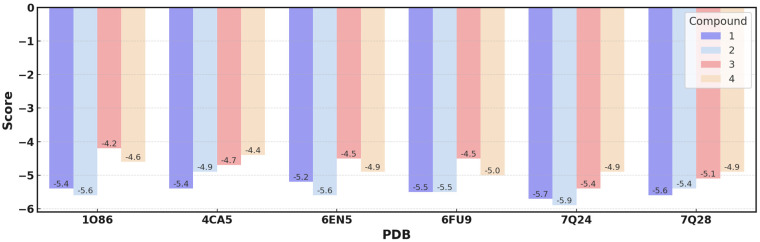
Graph of the affinity energies of the compounds studied the different Angiotensin-Converting Enzyme (ACE) receptor proteins. All values on the bar are expressed in energy units (kcal/mol).

Molecular docking was carried out using the parameters previously established during self-docking. This procedure was applied to each of the ligands under study, thus generating six results for each of them. In this way, it was possible to obtain a consensus regarding the positioning of each ligand. The results, represented in Figure 5, illustrate the affinity relationship of each ligand towards the ACE protein. The global average affinity energies are −5.1 kcal/mol, with specific values of −5.7, −5.5, −4.8, and −4.7 kcal/mol for **2**, **1**, **4**, and **3**, respectively. These results confirm the trend in biological activity, where it is observed that **2** is more active than the other three compounds under study.

**Figure 6:**
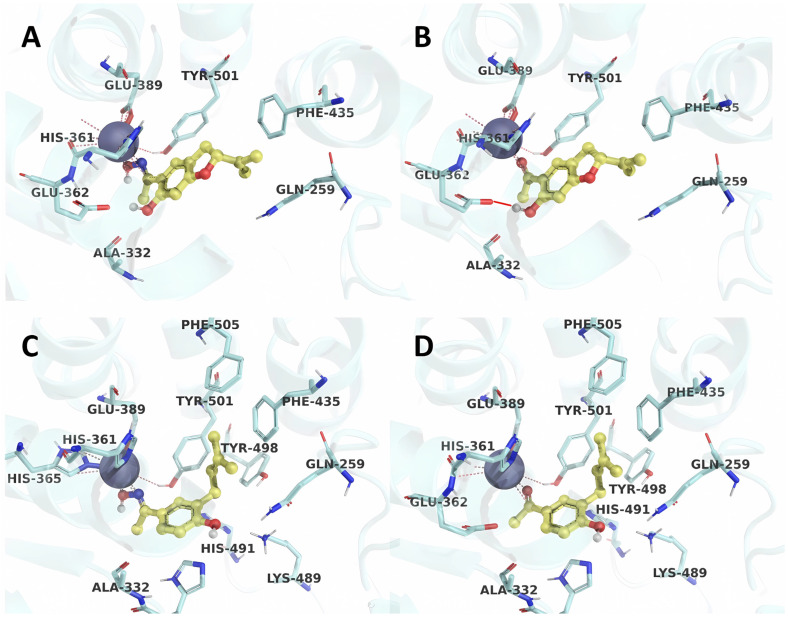
Interactions between the studied ligands with ACE in the binding site. The purple sphere represents the zinc atom, while compounds (**A**) **2**, (**B**) **1**, (**C**) **4**, and (**D**) **3** are shown in their respective poses obtained by molecular docking in the 7Q24 crystal as a representation of crystallized proteins.

As illustrated in Figure 6, the obtained poses highlight the interaction of an oxime group or carbonyls with the zinc atom, a feature shared with all ACE inhibitors. In particular, the compounds **2** and **1** stand out due to the presence of a hydroxyl group close to the electron-withdrawing group (oxime or carbonyl), which interacts with the Glu362 residue at a distance of 1.73 Å (Table 2). This interaction favors the arrangement of the ligand and its coordination with the zinc coordination sphere.

On the other hand, compounds **3** and **4**, which have longer side chains, present hydrogen bonds with distances greater than 3 Å (Table 2). These compounds, in turn, weakly influence ligand positioning due to the distances between the isopentyl side chain and residues Tyr501, Phe435, and Phe505 (Figure 6 and Table 3). This causes the ligand to have greater freedom when interacting at the binding site. In contrast, the most active compounds do not require an extensive aliphatic side chain due to the fixation they achieve through hydrogen bonding.

The analysis corroborates these results using the MM-GBSA method, which is used to calculate the binding energy, considering an implicit solvation in the calculation process (Table 4). The present analysis provides a detailed breakdown of the energetic contributions and highlights Coulomb energies and hydrogen bonding as the most preeminent energetic terms in the affinity of **2** and **1**, recording values of −1.33 and −1.20 kcal/mol, respectively. It should be noted that, despite the lack of emphasis on lipophilic and van der Waals contributions, these do not emerge as significant factors in the interactions between the various ligands examined.

## 3. Materials and Methods

### 3.1. Chemicals

Hydroxylamine hydrochloride, pyridine, magnesium sulfate, L-phenylephrine hydrochloride (PE), and acetylcholine chloride (ACh) were bought from Sigma-Aldrich (St. Louis, MO, USA). The metabolites and oximes were dissolved in DMSO (0.1% final concentration). The *S. nutans* extract was dissolved in a physiological solution.

### 3.2. Isolation of Natural Products from S. nutans and Oxime Synthesis

The natural product 5-acetyl-6-hydroxy-2-isopropenyl-2,3-dihydrobenzofurane (**1**) and 4-hydroxy-3-(isopenten-2-yl)-acetophenone (**3**) were isolated from *S. nutans* according to a previous protocol described elsewhere [11]. Briefly, the hydroalcoholic extract was resuspended in distilled water and extracted successively with *n*-hexane, dichloromethane (DCM), and ethyl acetate (EtOAc). Compounds **1** and **3** were isolated from dichloromethane subfraction. The organic solutions were concentrated on a rotary evaporator. The structural elucidation was carried out using spectroscopic data.

The synthesis of oximes (**2** and **4**) was performed as previously described with a few modifications [14,15]. To a solution of the keto-ester (500 mg, 2.5 mmol, 1.0) and hydroxylamine (180 mg, 2.5 mmol, 1.0), ethanol (10 mL) and pyridine (1.6 mL·mmol) were added in 1 portion. The reaction mixture was heated at 65 °C for 24 h and then concentrated on a rotary evaporator. The residue was partitioned between DCM (50 mL) and water (10 mL). The organic layer was sequentially washed with HCl (0.5 N) and water (10 mL) and then dried over anhydrous Na_2_SO_4_.

### 3.3. Animals

Male Sprague Dawley rats (6–8 weeks old; *n* = 12) weighing between 170 g and 200 g were used in this study. The investigation was conducted in accordance with the local animal research committee of Universidad de Antofagasta (CEIC #275/2020). The animals were housed in plastic cages at room temperature (22–25 °C) and a humidity of 45–51% and had full access to tap water and food (ad libitum). They were randomized and assigned into the different groups tested.

### 3.4. Isolation of Rat Aorta and Vascular Reactivity Assays

This procedure was performed based on the method previously described [14]. Animals were euthanized by cervical dislocation. The aortic rings were placed in organ bath with Krebs-Ringer bicarbonate (KRB) solution (in mM), containing 4.2 KCl, 1.19 KH_2_PO_4_, 120 NaCl, 25 NaHCO_3_, 1.2 MgSO_4_, 1.3 CaCl_2_, and 5 D-glucose, at pH 7.4, 37 °C, 95% O_2_, and 5% CO_2_. After an equilibration period of 30 min, the aortic rings were stabilized by 3 successive near-maximum contractions with KCl (60 mM) for 10 min. The passive tension on the aorta was 1.0 g, which was determined to be the resting tension for obtaining maximum active tension induced by 60 mM KCl [15]. The integrity of the vascular endothelium was assessed using 10^−5^ M acetylcholine (ACh) in precontracted aortic rings with 10^−6^ M phenylephrine (PE) at the beginning of the experiment. To evaluate the contractile response to angiotensin I (Ang I; 10^−6^ M), the tissue was pre-incubated with extract or compounds for 20 min prior to contraction. Ang I produces a transient contraction in the vasculature and only a single concentration was used due to the tachyphylaxis of the response [22].

### 3.5. Determination of Angiotensin I-Converting Enzyme (ACE) Activity

ACE activity assay was performed in vitro using a kit fluorometric from Sigma-Aldrich (CS0002; Merck; Darmstadt, Germany) to screen potential ACE inhibitors. A captopril inhibitor was used as a positive control for ACE activity. A fluorescence microplate reader equipped with Infinite 200 TECAN (Tecan Trading AGs, Männedorf, Switzerland) for excitation in the range of 320 nm and emission detection at 405 nm was used.

### 3.6. Preparation of Receptors and Ligands

Six crystalline complexes were obtained from the PDB database with a resolution of 2.0 Å or less (PDB ID 1O86 [23], 4CA5 [24], 6EN5 [25], 6F9U [26], 7Q24 [27], and 7Q28 [27]). These complexes extracted co-crystallized ligands and removed water molecules, salts, and other cofactors. The zinc atom was kept in the binding site, and its valence state was considered to be +2. The missing amino acids were added to each protein, the partial charges were incorporated, and the protonation of the corresponding basic and acid amino acids was performed at physiological pH. Finally, the side chains were relaxed using an OPLS4 force field [28]. The co-crystallized and synthesized ligands and the hydrogens were added and bond adjustments were carried out, considering the hybridization of each carbon atom. In addition, the grid of each complex was adapted to the size of its respective ligand, using the center of mass as a reference for the grid.

### 3.7. Molecular Docking

In this study, all crystalline complexes were aligned with the crystal structure 1O86. The coordinates for the center of the grid in this alignment were set at *X*: 41.01, *Y*: 34.33, and *Z*: 46.44. The dimensions of each grid were judiciously determined based on the size of the ligand co-crystallized with the respective complexes. The 7Q24 crystal was employed as a representative ACE receptor to interact with the molecules under investigation to conduct molecular docking. This docking process was carried out using the Glide program, utilizing the Standard Precision (SP) scoring function to evaluate the interactions [29,30].

To refine the initial results, affinity energy was determined using the MM-GBSA [31] calculation. This method is notable for considering the flexibility of amino acid side chains within the binding site. It also estimates the binding free energy, considering various factors such as solvation (∆GSolv_GB) and energetic contributions. These contributions respond to different interactions, including electrostatic (represented by Coulomb energy, ∆GCoul), hydrophobic (encompassed by van der Waals energy, ∆GvdW), hydrogen bond (∆GHbond), lipophilic (∆GLipo), and pi-stacking interactions (∆GPaking). The comprehensive calculations required for this analysis were executed using the Prime software (Prime, Version 2021-1 Schrödinger: New York, NY, USA, 2021) [30]. Finally, for the effective graphical visualization of the results, PyMOL (The PyMOL Molecular Graphics System, Version 2.0 Schrödinger, LLC) software was utilized, offering a clear and detailed representation of the molecular interactions and docking outcomes.

### 3.8. Statistical Analysis

The results obtained from the experiments are expressed as mean ± standard deviation of the mean (SD). Statistical analysis of the data was performed using analysis of variance (one- or two-way ANOVA) followed by the Bonferroni post hoc test. Graph Pad Prism software, version 5.0. (GraphPad Software, Inc., La Jolla, CA, USA), was used. Statistical significance was set at *p* < 0.05.

## 4. Conclusions

In conclusion, our investigation revealed that the oximes exhibited superior ACE inhibitory activity compared to the metabolites de *Senecio nutans*. Oxime **2** was the most potent compound in the series. Molecular docking calculations show that compound **2** presented a better profile of affinity and stability in the interaction with Gln259, Phe435, Tyr501, and Phe505 proteins. This study of the vascular contractile response to Angiotensin I showed that compound **2** significantly reduced Ang I contraction, underscoring its potential therapeutic impact. In contrast, **1** did not exhibit a comparable effect, consistent with the biological analysis. These comprehensive findings emphasize the promising therapeutic potential of **2** as an ACE inhibitor, paving the way for further exploration of *Senecio nutans* compounds in the context of hypertension treatment and vascular function modulation.

## Figures and Tables

**Table 1:** Self-docking results in relation to the docked pose with the reference co-crystallized ligand.

	1O86	4CA5	6EN5	6F9U	7Q24	7Q28
Score (kcal/mol)	−12.84	−14.21	−9.70	−11.62	−9.42	−11.02
MM-GBSA (kcal/mol)	−67.58	−89.32	−55.32	−78.48	−63.32	−69.67
RMSD (Å)	1.02	0.57	1.92	1.98	1.21	0.73

**Table 2:** Hydrogen bonds obtained with molecular docking.

	1	2	3	4
Gln259	-	-	3.20	3.04
Glu362	1.73	1.73	-	-
Tyr501	2.30	2.26	1.88	2.78

Values expressed in Å.

**Table 3:** Hydrophobic interactions obtained with molecular docking.

	1	2	3	4
Gln259	-	3.88	-	-
Phe435	3.28	3.23	3.5	3.5
Phe435	-	3.46	3.4	3.44
Tyr501	3.49	3.35	3.72	3.71
Tyr501	-	-	3.57	3.74
Phe505	-	3.84	3.41	3.38

Values expressed in Å.

**Table 4:** Energy contributions of the MM-GBSA calculation.

Entry	∆G_Bind_	∆G_Coul_	∆G_Hbond_	∆G_Lipo_	∆G_Packing_	∆G_Solv_GB_	∆G_vdW_
**1**	−39.99	−26.80	−1.20	−21.74	0.05	38.62	−32.26
**2**	−42.99	−29.05	−1.33	−21.50	−0.99	35.67	−30.38
**3**	−31.01	−19.07	−0.30	−22.23	1.07	37.60	−29.73
**4**	−34.80	−20.60	−0.50	−20.36	−0.22	30.35	−28.52

## Data Availability

Data is contained within the article.

## Abbreviations

The following abbreviations are used in this manuscript:

ACE	Angiotensin-Converting Enzyme
ACh	Acetylcholine chloride
Ang I	Angiotensin I
LTCC	L-type calcium channel
PE	L-phenylephrine hydrochloride
RAS	Renin–angiotensin system
